# A Liposomal Strategy for Dual-Action Therapy in Sarcopenia: Co-Delivery of Caffeine and HAMA

**DOI:** 10.3390/ijms26136031

**Published:** 2025-06-24

**Authors:** Alfred Najm, Alexandra Cătălina Bîrcă, Adelina-Gabriela Niculescu, Adina Alberts, Alexandru Mihai Grumezescu, Bianca Gălățeanu, Mircea Beuran, Bogdan Severus Gaspar, Claudiu Stefan Turculet, Ariana Hudiță

**Affiliations:** 1 Department of Surgery, Carol Davila University of Medicine and Pharmacy, 8 Eroii Sanitari, Sector 5, 050474 Bucharest, Romania; alfred.najm@yahoo.ro (A.N.); adina-magdalena.alberts@rez.umfcd.ro (A.A.); drmirceabeuran@yahoo.com (M.B.); bogdangaspar2005@yahoo.com (B.S.G.); claudiu.turculet@umfcd.ro (C.S.T.); 2 Emergency Hospital Floreasca Bucharest, 8 Calea Floreasca, Sector 1, 014461 Bucharest, Romania; 3 Department of Science and Engineering of Oxide Materials and Nanomaterials, National University of Science and Technology Politehnica Bucharest, 011061 Bucharest, Romania; ada_birca@yahoo.com (A.C.B.); adelina.niculescu@upb.ro (A.-G.N.); 4 Research Institute of the University of Bucharest—ICUB, University of Bucharest, 050657 Bucharest, Romania; bianca.galateanu@bio.unibuc.ro (B.G.); ariana.hudita@bio.unibuc.ro (A.H.); 5 Faculty of Biology, University of Bucharest, 050657 Bucharest, Romania

**Keywords:** sarcopenia, liposomal hybrid drug delivery system, caffeine, hyaluronic acid methacrylate

## Abstract

The biological complexity of sarcopenia presents a major challenge for therapeutic intervention due to the wide range of degenerative changes it induces in skeletal muscle. This study demonstrates the potential of liposomal controlled release systems to address these challenges by combining two bioactive agents with complementary actions: caffeine (CAF), encapsulated in DMPC-based liposomes, and hyaluronic acid methacrylate (HAMA), encapsulated in DOPC-based liposomes. A hybrid system was also developed to deliver both substances simultaneously, aiming to restore tissue function through combined metabolic, anti-inflammatory, and regenerative effects. The liposomes exhibited nanoscale dimensions, spherical morphology, and intact membrane structure, as confirmed by electron microscopy. DLS analysis indicated good colloidal stability and monodisperse size distribution across all formulations, with improved stability observed in the hybrid system. Drug release studies showed a time-dependent profile, with HAMA releasing rapidly and CAF releasing gradually, supporting a dual-action therapeutic approach tailored to the multifactorial pathology of sarcopenia. The biological assays, performed in an established in vitro sarcopenia model, revealed the potential of liposomes co-delivering caffeine and HAMA to mitigate oxidative stress, preserve mitochondrial function, and reduce apoptosis in H_2_O_2_-damaged myotubes.

## 1. Introduction

Numerous studies on skeletal muscle physiology have contributed to a comprehensive understanding of the structural complexity and multifunctional roles of this tissue, particularly in terms of contraction, movement, and force generation. Within the category of striated muscles, two primary types are recognized: cardiac and skeletal muscles. Cardiac muscle is composed of self-stimulating, non-fatiguing myocytes with moderate energy demands, designed for continuous rhythmic activity. In contrast, skeletal muscles consist of voluntarily innervated fibers, which are susceptible to fatigue and are characterized by high metabolic and energy requirements. Skeletal muscles serve as a primary reservoir of amino acids derived from both dietary intake and physical activity, playing a central role in protein storage and muscle hypertrophy [1,2,3]. However, this tissue is subject to various degenerative changes, some of which are age-related, while others occur independently of chronological aging. In such cases, the condition is clinically diagnosed as sarcopenia.

Sarcopenia is an increasingly prevalent and alarming syndrome characterized by the progressive and accelerated loss of muscle mass and strength [3,4]. As muscular atrophy and weakness develop, individuals experience reduced mobility, an increased risk of falls and fractures, and a decline in quality of life. Psychologically, this leads to a fear of movement, particularly a fear of falling, further exacerbating physical decline and social isolation [5]. Sarcopenia is a multifactorial condition influenced by a combination of intrinsic and extrinsic factors. Aging and genetic predispositions affect muscle metabolism and individual susceptibility [6]. Hormonal imbalances, particularly the age-related decline in anabolic hormones such as testosterone, growth hormone, and estrogen, play a crucial role alongside chronic low-grade inflammation commonly observed in elderly individuals [7]. Lifestyle aspects, including poor nutrition, physical inactivity, smoking, and excessive alcohol intake, also contribute significantly to muscle deterioration [8]. Moreover, chronic diseases like diabetes, cardiovascular conditions, and mobility-limiting illnesses further exacerbate muscle loss through systemic metabolic dysregulation and reduced activity levels [9].

**Figure 1:**
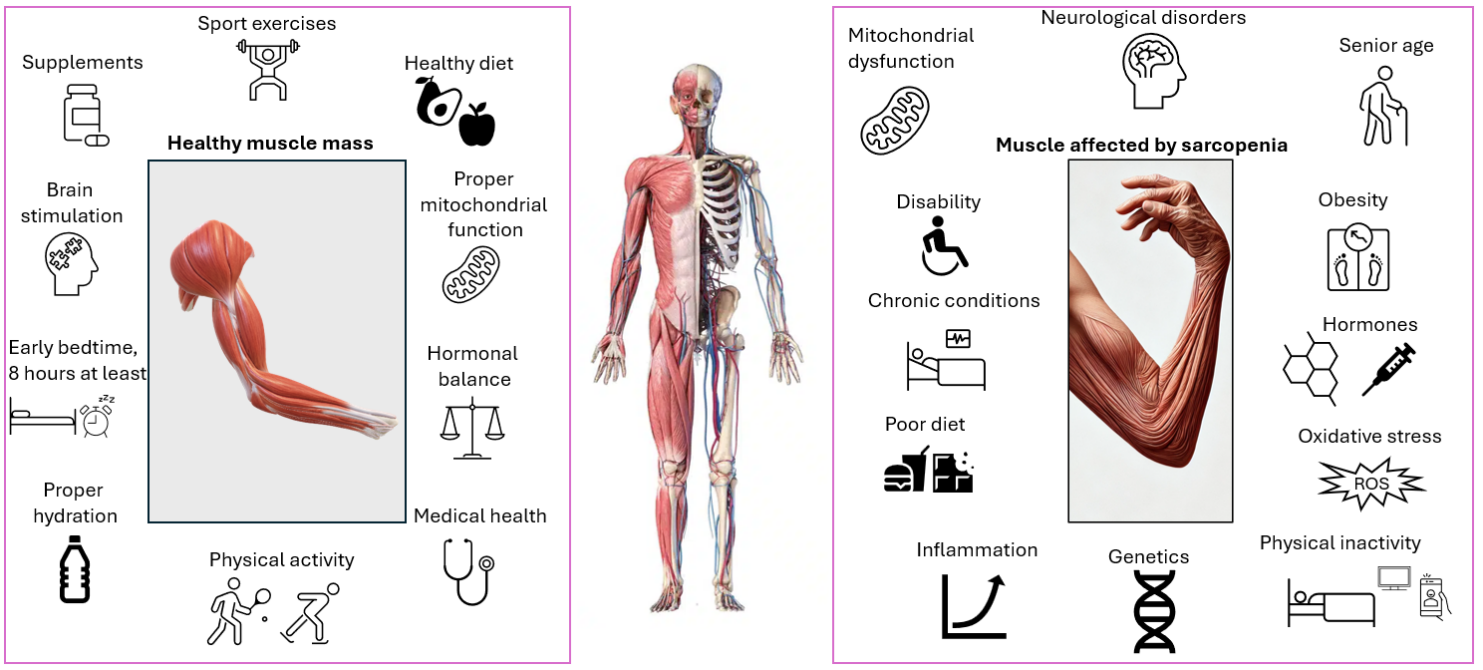
The conditions necessary to maintain healthy skeletal muscle tissue and the factors that influence and are found in the case of sarcopenia.

From a pathophysiological standpoint, sarcopenia results from a complex interplay of biological disruptions, often based on a mix of factors. These include alterations in muscle architecture, hormonal imbalances, genetic predispositions, chronic illnesses, systemic inflammation, nutritional deficiencies, and physical inactivity (Figure 1) [10,11,12,13]. In sarcopenic muscle tissue, significant cellular changes are observed. There is a reduction in the size and number of type II muscle fibers, which are crucial for strength and power. Additionally, the population of satellite cells, the muscle-resident stem cells responsible for muscle repair and regeneration, are both diminished in number and functionally impaired [14].

A characteristic protein imbalance is also evident in sarcopenia, with increased protein degradation, often driven by oxidative stress, leading to a net protein deficit and consequent loss of muscle strength and mass [8]. Another defining feature is the presence of chronic low-grade inflammation, marked by elevated plasma levels of pro-inflammatory cytokines. These cytokines have been shown to negatively impact skeletal muscle protein synthesis, interfere with growth hormone signaling, and accelerate muscle catabolism [7,15,16]. Moreover, elevated oxidative stress is frequently observed in sarcopenic individuals. This condition is closely associated with mitochondrial dysfunction and is believed to contribute to the early onset and progression of sarcopenia by exacerbating cellular damage and impairing metabolic homeostasis [17].

The biological disruptions observed in sarcopenia are highly complex and interdependent. When analyzed in correlation, these alterations reveal a multifactorial cascade of dysfunctions, particularly affecting skeletal muscle tissue at the cellular level. These cumulative changes underscore the urgency of identifying targeted and efficient therapeutic strategies. Currently, no universally approved pharmacological treatment exists for sarcopenia, but several therapeutic strategies are under investigation, often combining pharmaceutical and lifestyle-based interventions such as tailored nutrition and physical activity. A protein-rich diet, particularly with leucine, an essential amino acid, has been shown to support muscle protein synthesis. The co-administration of vitamin D, especially in elderly individuals with deficiencies, further enhances muscle strength. Additional dietary components under investigation for their myoprotective or regenerative roles include omega-3 fatty acids, HMB (beta-hydroxy-beta-methylbutyrate), creatine, arginine, and various antioxidants (e.g., vitamins C and E, coenzyme Q10), alongside foods like spinach, beetroot juice, and arugula. Anti-inflammatory nutraceuticals such as curcumin, resveratrol, green tea extract, and ginkgo biloba are also being explored for their roles in reducing muscle catabolism and promoting healthy aging. On the pharmacological front, several agents remain in experimental stages, including testosterone, anabolic steroids, growth hormone, insulin-like growth factor-1 (IGF-1), and ghrelin analogs. More targeted therapies, such as selective androgen receptor modulators (SARMs) and myostatin inhibitors, are showing promise in clinical trials. Additionally, NAD^+^ precursors like nicotinamide mononucleotide (NMN) are being investigated for their ability to counteract mitochondrial dysfunction and chronic inflammation, offering a novel cellular-level therapeutic pathway for sarcopenia management [4,18,19,20,21,22].

Sarcopenia today lacks approved pharmacological treatment, with existing approaches largely limited to nutritional support and physical exercise, despite extensive research and numerous clinical trials. According to a report published by the International Conference on Frailty and Sarcopenia Research (ICFSR), one of the main challenges in developing effective treatments lies in the complex and multifactorial nature of the disease. Unlike traditional medical conditions that often involve a single pathological pathway, sarcopenia encompasses a broad spectrum of physiological alterations. Additionally, the clinical presentation varies significantly among patients; while some may exhibit only two contributing factors, others may suffer from a combination of age-related comorbidities, complicating treatment strategies. In this context, the pharmaceutical approach proposed in the present study involves bioactive compounds encapsulated in biocompatible lipid vesicles. Compared to numerous treatments under investigation, this strategy offers a safer and less invasive alternative that can be administered locally without having invasive effects on patients. However, given sarcopenia’s multifactorial pathogenesis, extensive and prolonged testing is essential for validating potential pharmacotherapies. Moreover, global research efforts have also been disrupted by external factors such as the COVID-19 pandemic, which redirected scientific focus and temporarily halted progress in unrelated medical fields, including sarcopenia research [23,24,25].

In this context, the present study introduces a novel pharmacotherapeutic approach aimed at the prevention, amelioration, and treatment of sarcopenia. The proposed strategy involves developing and testing controlled drug delivery systems based on liposomal structures, in which therapeutic agents are encapsulated to ensure sustained and targeted action. The choice of a liposome-based system is grounded in its numerous advantages over conventional delivery platforms. Primarily, the phospholipid bilayer of liposomes closely mimics the natural architecture of cellular membranes, enabling efficient interaction, uptake, and fusion with target cells. Furthermore, liposomes exhibit high biocompatibility and are inherently non-immunogenic, making them suitable for repeated or systemic administration. Their self-assembly capability, along with the ability to encapsulate a wide range of hydrophilic and lipophilic molecules, enhances their therapeutic versatility. Additionally, liposomes are biodegradable and are eliminated from the body without exerting nephrotoxic effects, which is a critical factor in ensuring safety, particularly in vulnerable populations [26,27,28,29].

To address the multifactorial biological alterations associated with sarcopenia, two distinct phospholipids were selected as the basis for liposome formulation: 1,2-dimyristoyl-sn-glycero-3-phosphocholine (DMPC) and 1,2-dioleoyl-sn-glycero-3-phosphocholine (DOPC). These phospholipids were used to encapsulate two bioactive compounds with therapeutic potential against the cellular and molecular disruptions observed in sarcopenic muscle. Accordingly, a hybrid liposomal system was developed and tested, consisting of DMPC-based liposomes encapsulating caffeine (CAF), and DOPC-based liposomes encapsulating hyaluronic acid methacrylate (HAMA).

The choice of caffeine as a therapeutic agent was guided by its well-documented antioxidant, anti-inflammatory, and antidiabetic properties. As the principal active compound in one of the most widely consumed beverages globally, coffee, CAF has demonstrated beneficial effects relevant to sarcopenia management. Several studies involving human subjects who consumed coffee under clinical supervision reported improved muscle function and enhanced activity of satellite cells, both critical factors in counteracting sarcopenia [30,31]. Furthermore, experimental studies in aged mice revealed that regular coffee intake resulted in significantly greater muscle strength and muscle mass compared to control groups [30,32]. Mitochondrial dysfunction is a key hallmark of sarcopenia, contributing to a cascade of degenerative processes within skeletal muscle. In this context, CAFdemonstrates therapeutic potential by promoting autophagy (autophagosome), a complex process that completes the formation of double-membrane vesicles on molecules that have been declared to experience degradation. Moreover, CAFstimulates mitochondrial biogenesis in skeletal muscle by activating AMP-activated protein kinase (AMPK) and peroxisome proliferator-activated receptor-gamma coactivator 1-alpha (PGC-1α), two critical regulators of cellular energy metabolism and mitochondrial function [33,34,35]. Additionally, skeletal muscle functions as an endocrine organ, releasing cytokines, known as myokines, during contraction. Among these, interleukin-6 (IL-6) plays a central role in energy balance and metabolic regulation. Notably, CAFadministration has been shown to mimic the effects of physical exercise by inducing IL-6 production in skeletal muscle, further supporting its role in muscle homeostasis and systemic metabolic activation [36,37].

On the other hand, hyaluronic acid (HA) is a naturally occurring glycosaminoglycan and a fundamental component of the extracellular matrix in various tissues, including skeletal muscle. In muscle tissue, hyaluronate synthesis is mediated by the enzymes hyaluronate synthases 1–3, which are essential for the differentiation of myoblasts into mature myofibers, a process intricately linked to the activity of satellite cells. Adequate HAavailability is crucial for maintaining the integrity of the muscle stem cell niche and supporting healthy muscle tissue remodeling [38,39,40]. In sarcopenia, this regenerative capacity is severely impaired, with a notable reduction in satellite cell function and failure of normal muscle maintenance and repair processes. Restoring HAlevels in a sarcopenic muscle through natural biosynthesis alone is a slow and often insufficient process, particularly when relying solely on physical activity to stimulate endogenous production. Therefore, exogenous supplementation becomes essential to replenish the extracellular matrix and re-establish the muscle stem cell niche. The presence of HAin the tissue compensates for what is deficient, thereby supporting cellular communication and regeneration.

Furthermore, several studies have shown that HAadministration initiates anti-inflammatory effects. For instance, in horse models, HAtreatment alleviated lameness, while in human clinical settings, intra-articular HA injections have demonstrated a reduction in TNF-α production via CD44 receptor-mediated signaling within the synovial fluid. Similar anti-inflammatory pathways are proposed to be active in skeletal muscle, where HA protects resident cells from molecular damage caused by oxidative and inflammatory stress [39,41,42].

**Scheme 1:**
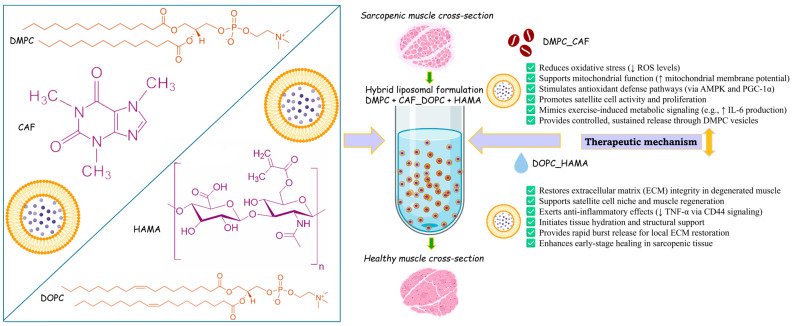
Schematic representation of the hybrid liposomal formulation design and therapeutic mechanism.

Therefore, this study proposes and evaluates a liposomal therapeutic strategy (Scheme 1) designed to address the complex pathological mechanisms of sarcopenia through a multifactorial approach.

## 2. Results

**Figure 2:**
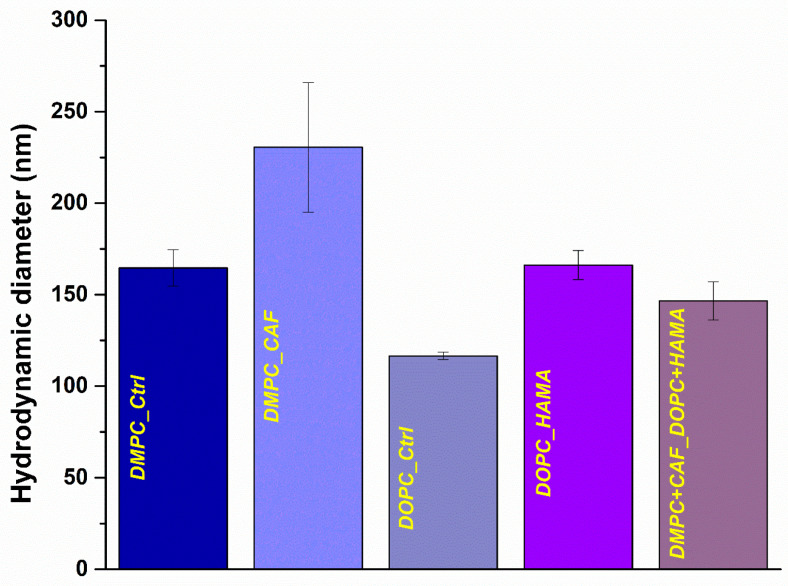
DLS results as hydrodynamic diameter (nm) registered for DMPC_Ctrl, DMPC_CAF, DOPC_Ctrl, DOPC_HAMA, and DMPC + CAF_DOPC + HAMA.

The experimental results were obtained from investigations conducted on five liposomal formulations synthesized in the laboratory. These included two control samples, DMPC_Ctrl and DOPC_Ctrl, along with three active formulations: DMPC_CAF, consisting of DMPC-based liposomes encapsulating CAF; DOPC_HAMA, comprising DOPC-based liposomes encapsulating HAMA; and a hybrid formulation, DMPC + CAF_DOPC + HAMA, which combined both lipid types and active compounds. These variations in lipid composition and encapsulated agents were strategically designed to assess potential differences in physicochemical behavior, allowing for comparative analysis across all samples. In the first characterization phase, the liposomal formulations were analyzed using the DLS technique to evaluate their suspension properties, including hydrodynamic diameter, zeta potential, and polydispersity index. Figure 2 presents a graphical comparison of the hydrodynamic diameter values (expressed in nanometers) obtained for each of the five formulations, while Table 1 provides the corresponding numerical data for enhanced clarity and quantitative analysis.

Regarding the hydrodynamic diameter values obtained for liposomal formulations, noticeable differences were observed among the control samples, depending on the type of phospholipid used. The DMPC_Ctrl sample exhibited a larger average diameter (164.63 nm) compared to the DOPC_Ctrl sample (116.5 nm), indicating that the lipid composition significantly influences the size of the resulting liposomes. Furthermore, the incorporation of active substances led to an increase in hydrodynamic diameter for both lipid types when compared to their respective controls, with DMPC_CAF (230.53 nm) displaying a larger average diameter than DOPC_HAMA (166.2 nm), suggesting that the encapsulated agent and lipid type together influence liposome size. Interestingly, the hybrid formulation (DMPC + CAF_DOPC + HAMA) exhibited a reduced hydrodynamic diameter relative to the individual therapeutic liposomes. This observation may be attributed to potential interactions between the two phospholipid types during formulation. Although equal volume ratios were used in the mixture, it is possible that the DOPC_HAMA formulation contributed to a higher number of liposomes, thereby leading the DLS measurement toward smaller diameters. As DLS analysis is intensity-weighted, the dominance of one population, particularly smaller or more numerous vesicles, can significantly influence the reported average size.

**Figure 3:**
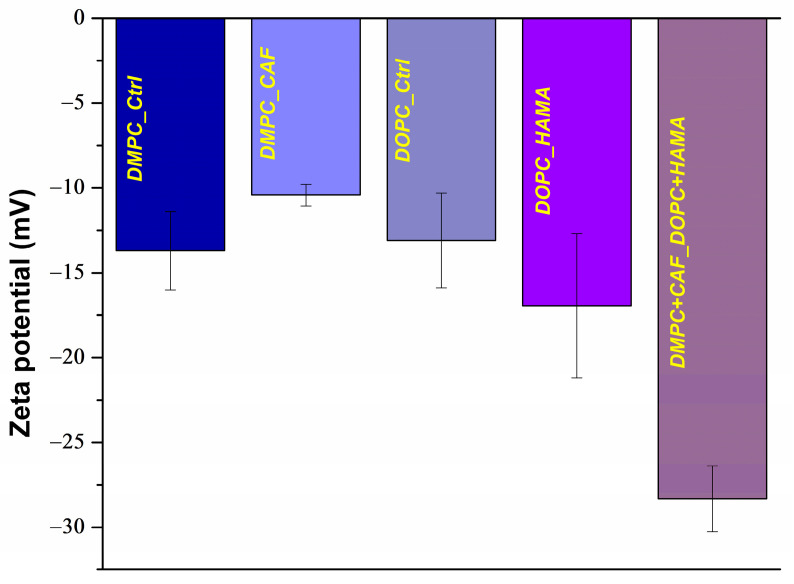
DLS result as zeta potential (mV) registered for DMPC_Ctrl, DMPC_CAF, DOPC_Ctrl, DOPC_HAMA, and DMPC + CAF_DOPC + HAMA.

Another key parameter assessed through DLS was the zeta potential of the formulated liposomes. The results are illustrated in Figure 3 and detailed in Table 2. The control samples, DMPC_Ctrl and DOPC_Ctrl, exhibited comparable zeta potential values of approximately −13 mV, indicating a baseline level of electrostatic repulsion and moderate colloidal stability in aqueous suspension. Upon encapsulation of therapeutic agents, changes in surface charge were observed. The DMPC_CAF formulation recorded an average zeta potential of −10.42 mV, while DOPC_HAMA exhibited a more negative value of −16.94 mV, both suggesting moderate stability in suspension, but with slight shifts in charge likely attributed to the physicochemical interactions between the encapsulated molecules and the lipid bilayer. In contrast, the hybrid formulation, DMPC + CAF_DOPC + HAMA, demonstrated a substantially more negative zeta potential of −28.32 mV, indicative of enhanced stability. This suggests that the co-existence and interaction of the two types of liposomes may contribute to a more stabilized system, possibly due to electrostatic equilibrium or bilayer reorganization that promotes better dispersion and reduced aggregation in suspension.

All formulations exhibited a polydispersity index (PDI) of approximately 0.2 (Table 3), indicating that the liposomal suspensions were monodisperse with a uniform size distribution. This value reflects a high degree of homogeneity in liposome size, which is a desirable characteristic for liposome-based drug delivery systems. Accordingly, liposomes can be considered to possess acceptable colloidal stability.

**Figure 4:**
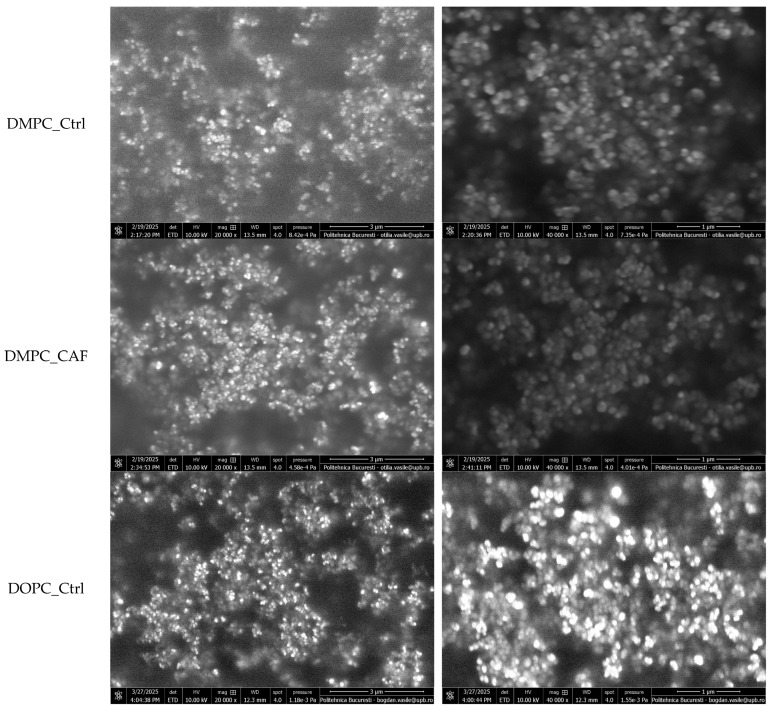

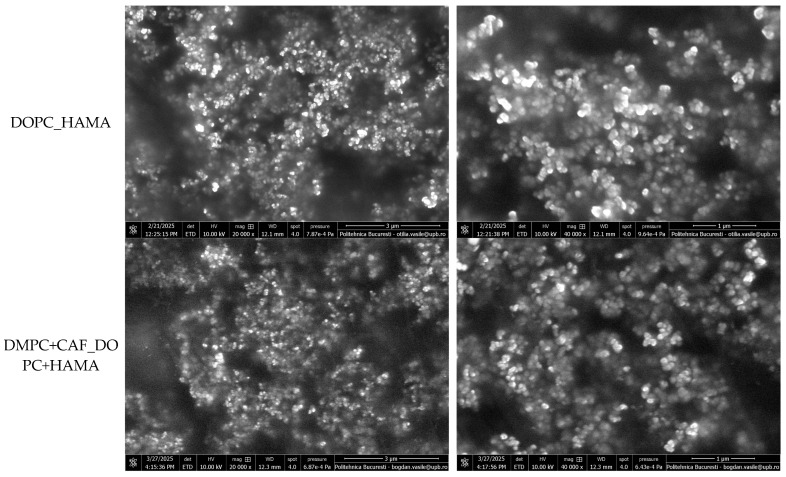
SEM micrographs (20,000×, 40,000×) obtained for DMPC_Ctrl, DMPC_CAF, DOPC_Ctrl, DOPC_HAMA, and DMPC + CAF_DOPC + HAMA.

To further characterize the morphology and surface structure of the liposomal formulations, SEM was conducted. Representative micrographs, acquired at 20,000× and 40,000× magnification, are shown in Figure 4.

All tested samples exhibited highly similar morphological features, confirming the reproducibility of the synthesis protocol. The SEM images revealed that the liposomes display a spherical and well-defined vesicular architecture, typical of classical liposomal systems. The vesicles appear smooth-surfaced and intact, suggesting structural stability and the absence of visible defects, ruptures, or fusion events. A moderate degree of vesicle clustering and surface contact was observed, likely due to drying-induced agglomeration during sample preparation. However, individual boundaries remained distinguishable, indicating that liposomes retained their discrete morphology.

**Figure 5:**
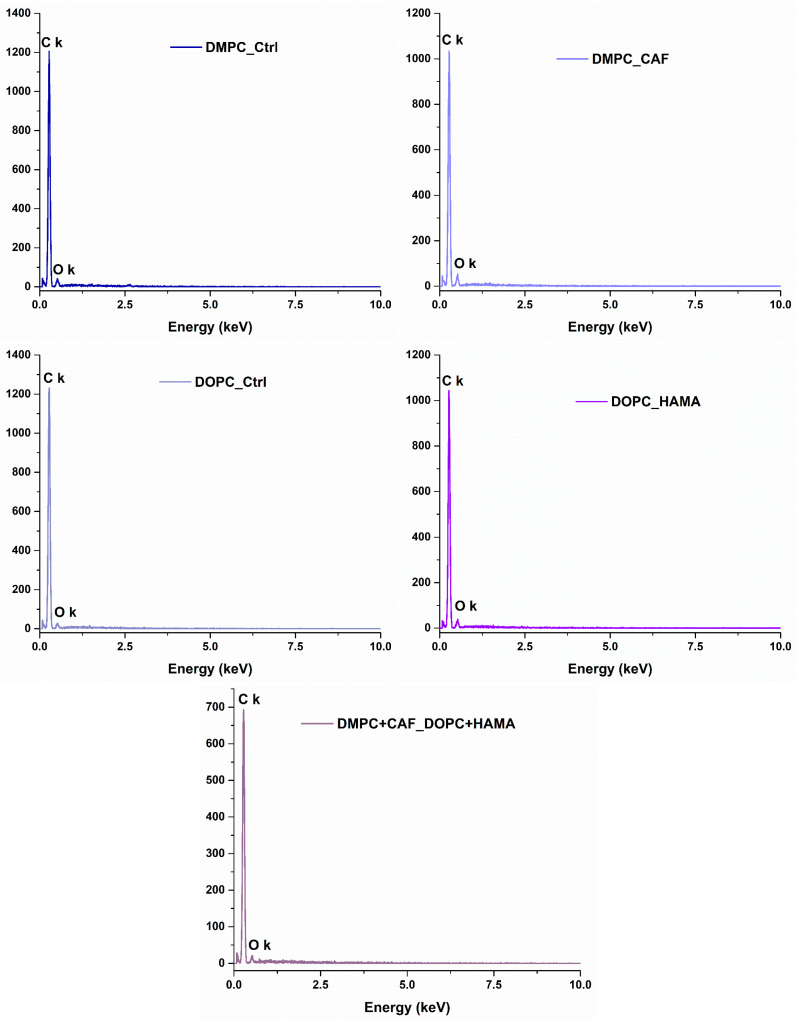
EDS results obtained for DMPC_Ctrl, DMPC_CAF, DOPC_Ctrl, DOPC_HAMA, and DMPC + CAF_DOPC + HAMA.

From an elemental composition perspective, the therapeutic liposomes exhibited consistent chemical profiles across all formulations. Using the EDS module, the primary elements identified were carbon (C) and oxygen (O), both expected components of the organic molecular structure of the phospholipid-based systems. The absence of additional elemental signals confirms the purity of the samples and the lack of inorganic or contaminant residues. These findings support the conclusion that all liposomal formulations are compositionally similar at the elemental level, reinforcing the reproducibility and cleanliness of the synthesis process. The EDS results are illustrated in Figure 5.

**Figure 6:**
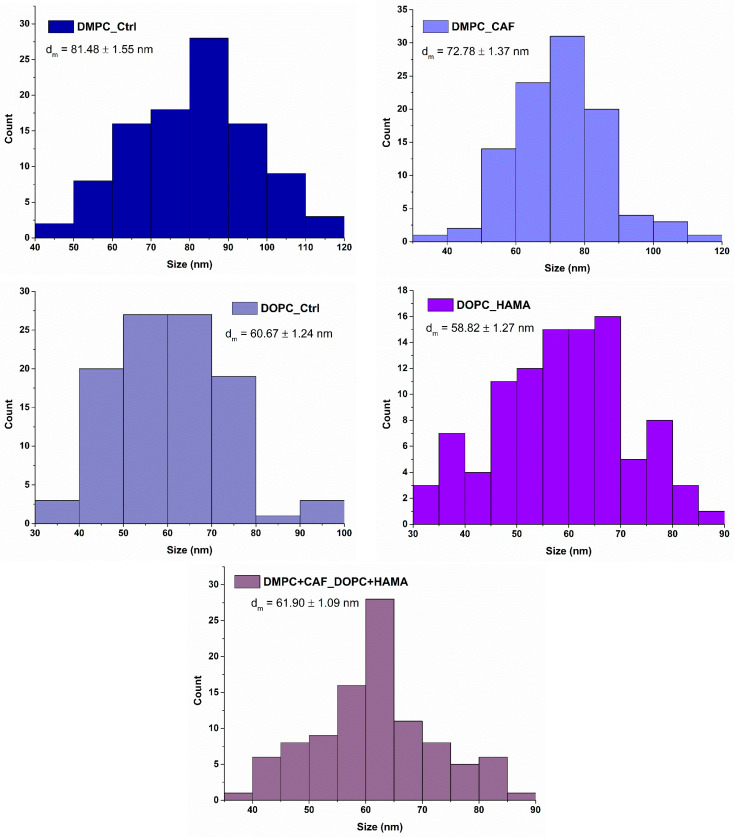
Liposome size distributions obtained for DMPC_Ctrl, DMPC_CAF, DOPC_Ctrl, DOPC_HAMA, and DMPC + CAF_DOPC + HAMA.

To evaluate the average size of the synthesized liposomes, measurements were performed based on the acquired SEM micrographs. The data was used to generate size distribution histograms, reflecting the nanometric dimensions of the liposomal vesicles. These results are presented in Figure 6.

The average vesicle sizes were determined from the SEM-based measurements, revealing trends that are generally consistent with the hydrodynamic diameter results obtained via DLS. Dimensional differences were observed between the control formulations, with DMPC-based liposomes exhibiting larger average diameters than those formed with DOPC. Specifically, the DMPC_Ctrl sample showed an average size of 81.48 ± 1.55 nm, while the DOPC_Ctrl sample measured 60.67 ± 1.24 nm. Interestingly, in contrast to the DLS data, where encapsulation of the active substances resulted in an increase in hydrodynamic diameter, the physical sizes measured using SEM results were slightly reduced following drug encapsulation. This observation may reflect changes in surface structure or compaction of the lipid bilayer during formulation. In the case of the hybrid formulation, the results further support the hypothesis that, despite equal volume mixing, the DOPC_HAMA population may be more abundant or dominant. The average vesicle size of the hybrid sample was more closely aligned with that of DOPC_HAMA, reinforcing the idea that the physical characteristics of the DOPC-based liposomes influence the overall size distribution within the hybrid system.

**Figure 7:**
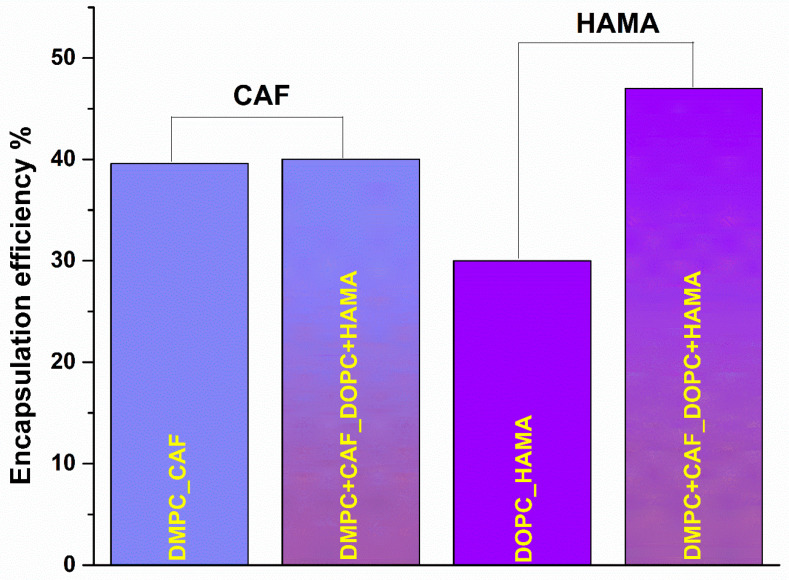
Encapsulation efficiency of caffeine and HAMA, evaluated for DMPC_CAF, DOPC_HAMA, and DMPC + CAF_DOPC + HAMA.

The comprehensive evaluation of liposomal properties, including hydrodynamic diameter, physical size, zeta potential, dispersion stability, and morphology, confirmed that the obtained formulations possess physicochemical characteristics well-suited for biomedical application, consistent with values typically observed in classical liposomal systems. Following this characterization, the next step was to assess the therapeutic potential of the liposomes by determining their encapsulation efficiency (EE%), a key parameter that not only reflects formulation efficiency but also correlates with physicochemical stability and the controlled release profile of the active compounds under physiologically simulated conditions. As illustrated in Figure 7, the encapsulation efficiency was evaluated for CAF and HAMA in both individual formulations DMPC_CAF and DOPC_HAMA as well as in the hybrid formulation (DMPC + CAF_DOPC + HAMA). The hybrid system was analyzed with respect to each encapsulated agent, showing comparable encapsulation percentages for CAF and HAMA relative to their individual formulations.

The encapsulation efficiency of CAF and HAMA was quantified to evaluate the drug-loading capacity of the liposomal systems. The results indicate that CAF exhibited an EE of 39.6% in the DMPC_CAF formulation, while the hybrid formulation (DMPC + CAF_DOPC + HAMA) achieved a slightly higher encapsulation efficiency of 40.6%. In contrast, HAMA showed an EE of 30% in the DOPC_HAMA formulation, which increased to 47% in the hybrid formulation. These findings suggest that CAF and HAMA exhibit distinct encapsulation behaviors, likely influenced by the physicochemical properties of both the phospholipids and the active substances. CAF demonstrated relatively stable encapsulation in single and mixed systems, consistent with its compatibility with DMPC-based liposomes. HAMA, on the other hand, showed a notably improved encapsulation efficiency in the hybrid system.

**Figure 8:**
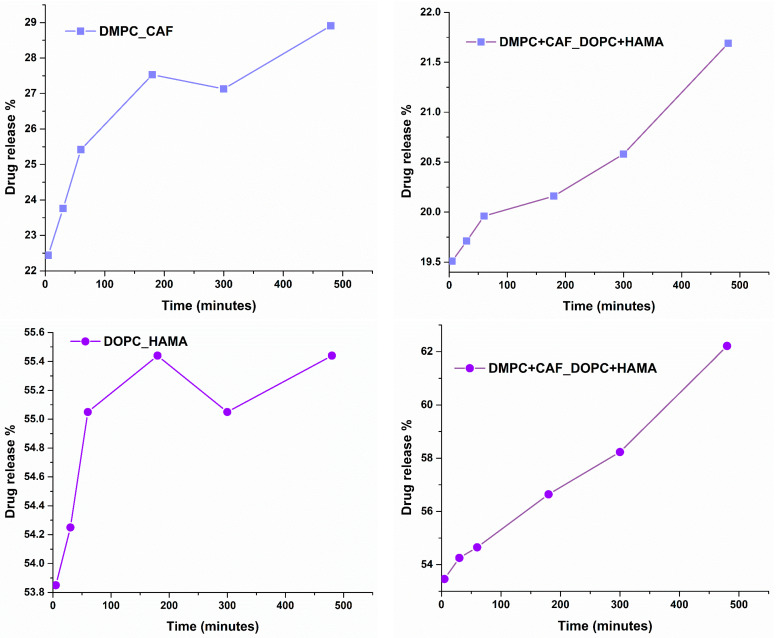
Drug release rate of caffeine and HAMA evaluated for DMPC_CAF, DOPC_HAMA, and DMPC + CAF_DOPC + HAMA depending on time (8 h = 480 min).

An essential parameter in developing liposomal drug delivery systems is the time-dependent release profile of the encapsulated therapeutic agents. To evaluate this, the release rates of CAF and HAMA were assessed in a physiologically relevant medium (PBS) at 37 °C, simulating in vivo conditions. The release was monitored over an 8 h period, providing insights into the kinetics of drug release and its potential correlation with therapeutic efficacy. Figure 8 presents the release profiles of CAF and HAMA from their respective individual liposomal formulations (DMPC_CAF and DOPC_HAMA), as well as from the hybrid formulation (DMPC + CAF_DOPC + HAMA) for dual-component delivery.

The release behavior of active substances is notably influenced by the physicochemical properties of the phospholipids used in liposomal formulations. In the case of DMPC_CAF, the release profile of CAF demonstrates a gradual, time-dependent pattern, starting at approximately 22% and increasing steadily to nearly 29% over the course of 8 h. This reflects controlled and sustained release behavior, which is characteristic of DMPC-based systems. Conversely, the DOPC_HAMA formulation shows a more immediate release profile, with an initial release of approximately 53.8%, reaching 55.5% by the end of the 8 h period. This suggests a burst release effect, followed by a slower release plateau, likely due to the more fluid and permeable nature of the DOPC lipid bilayer. In the case of the hybrid formulation (DMPC + CAF_DOPC + HAMA), the release profiles of both compounds remain consistent with those observed in their respective individual systems. For CAF, the hybrid system shows a release increase from approximately 19.5% to 21.6%, while HAMA shows a continued release from 54% to 62% over the same timeframe. These results suggest that the co-existence of the two lipid types in the hybrid formulation does not compromise the release characteristics of either agent.

**Figure 9:**
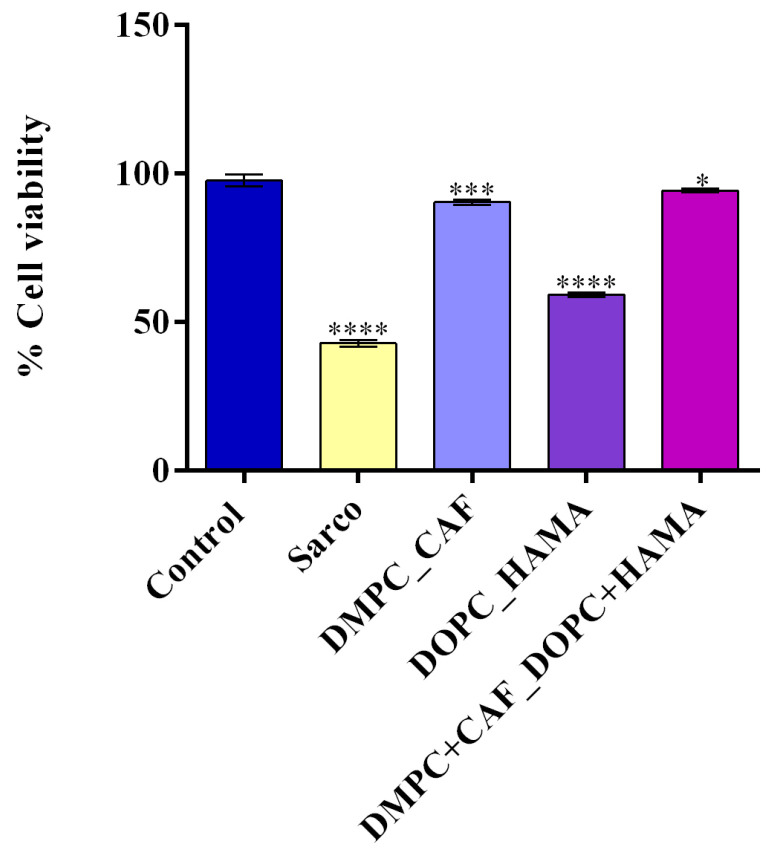
Viability of C2C12 myotubes assessed by MTT assay after 48 h of treatment with DMPC_CAF, DOPC_HAMA, and the combined DMPC + CAF_DOPC + HAMA formulation. Except for the experimental control (untreated C2C12 myotubes), all groups were exposed to H_2_O_2_ to induce a sarcopenia-like phenotype. Data are expressed as mean ± standard deviation (SD). Statistical analysis was performed using GraphPad Prism 6 software. Significance levels compared to the control group are indicated as follows: *p* ≤ 0.05 (*), *p* ≤ 0.001 (***), and *p* ≤ 0.0001 (****).

Following the physicochemical characterization of the liposomal formulations, their biological effects were evaluated in vitro using differentiated C2C12 myotubes exposed to H_2_O_2_-induced oxidative stress, thereby establishing a cellular model for sarcopenia. To assess the cytotoxic effects of H_2_O_2_-induced oxidative damage and the potential protective effects of liposome-encapsulated bioactive compounds, an MTT assay was performed (Figure 9). Treatment with H_2_O_2_ for 48 h (Sarco group) led to a statistically significant decrease in cell viability compared to the untreated control, confirming the successful induction of a sarcopenia-like phenotype characterized by impaired cell survival due to oxidative stress. The liposomal treatments tested revealed varying degrees of cytoprotective effects against the oxidative damage induced by H_2_O_2_ treatment. After 48h of treatment with DOPC_HAMA, C2C12 myotubes exhibited a minor recovery in cell viability, as cell viability remained significantly lower than that of the control group (**** *p* ≤ 0.0001). Treatment with DMPC_CAF triggered a partial recovery of cell viability, with a significantly pronounced effect greater than that of DOPC_HAMA, as cell viability was significantly higher compared to both the Sarco group (**** *p* ≤ 0.0001) and the DOPC_HAMA group (**** *p* ≤ 0.0001). Notably, the most substantial improvement was observed in the DMPC + CAF_DOPC + HAMA group, where cell viability approached control levels, suggesting a possible synergistic interaction between the bioactive compound cargos.

**Figure 10:**
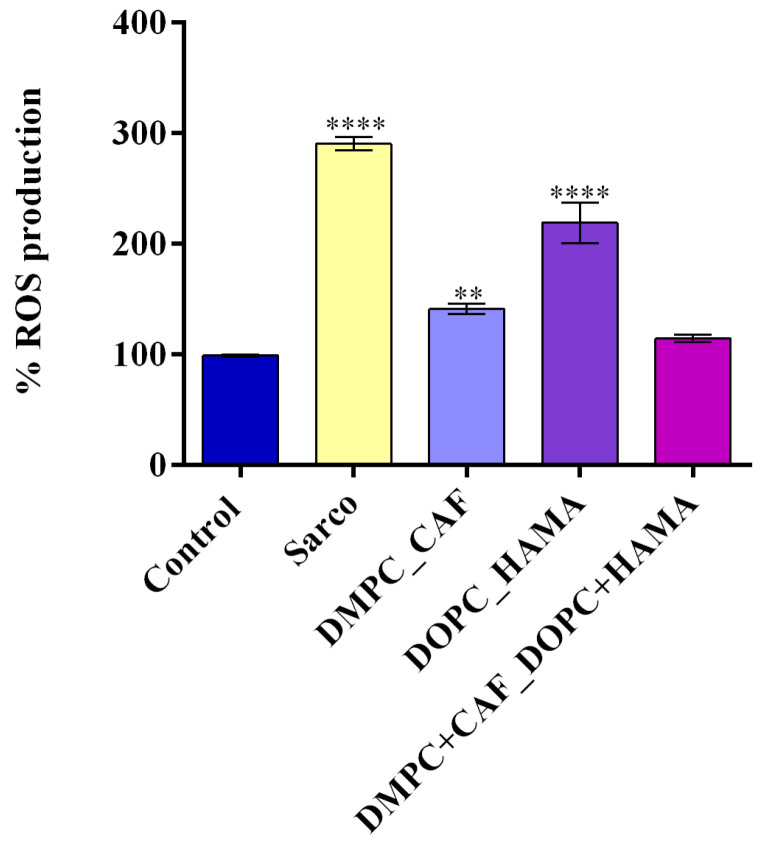
Intracellular ROS levels in C2C12 myotube cultures after 48 h of treatment with DMPC_CAF, DOPC_HAMA, and the combined DMPC + CAF_DOPC + HAMA formulation. Except for the experimental control (untreated C2C12 myotubes), all groups were exposed to H_2_O_2_ to induce a sarcopenia-like phenotype. Data are expressed as mean ± standard deviation (SD). Statistical analysis was performed using GraphPad Prism. Significance levels compared to the control group are indicated as follows: *p* ≤ 0.01 (**) and *p* ≤ 0.0001 (****).

Furthermore, oxidative stress, recognized as a hallmark of sarcopenia, was evaluated to assess the ability of liposome-encapsulated bioactive compounds to mitigate H_2_O_2_-induced oxidative damage after 48h of treatment. As shown in Figure 10, the Sarco group displayed statistically significant elevated intracellular ROS levels compared to the experimental control (**** *p* ≤ 0.0001), results that confirm the induction of oxidative damage in C2C12 myotube cultures after H_2_O_2_ treatment. Treatment with DMPC_CAF led to a pronounced reduction in ROS levels relative to the Sarco group (**** *p* ≤ 0.0001), indicating strong antioxidant activity of caffeine-loaded liposomes. However, ROS levels under DMPC_CAF treatment remained significantly elevated compared with the experimental control (** *p* ≤ 0.01). Conversely, DOPC_HAMA treatment produced a moderate decrease in ROS levels compared with the Sarco group (*** *p* ≤ 0.001), while oxidative stress remained significantly higher compared with the experimental control (**** *p* ≤ 0.0001), suggesting a limited antioxidant potential of HAMA-loaded liposomes under these conditions. Notably, the combined DMPC + CAF_DOPC_HAMA formulation significantly suppressed ROS generation, nearly restoring intracellular ROS levels to those quantified in the experimental control. These findings highlight a potential synergistic antioxidant effect resulting from the co-delivery of CAF and HAMA, reinforcing the potential of the tested liposomal encapsulation in combating oxidative injury in sarcopenia-like models.

**Figure 11:**
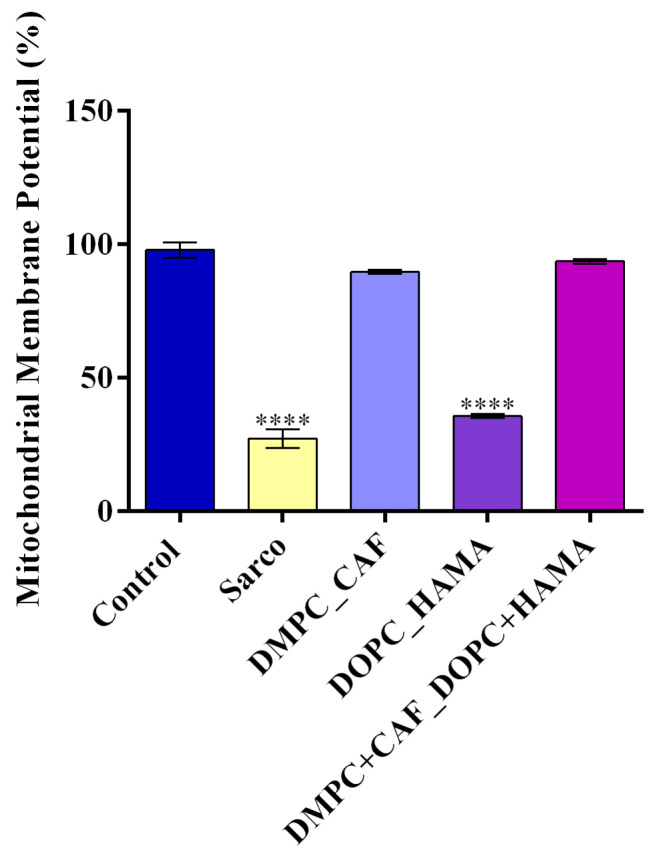
Mitochondrial membrane potential (MMP) in C2C12 myotubes cultures after 48 h of treatment with DMPC_CAF, DOPC_HAMA, and the combined DMPC + CAF_DOPC + HAMA formulation. Except for the experimental control (untreated C2C12 myotubes), all groups were exposed to H_2_O_2_ to induce a sarcopenia-like phenotype. Data are expressed as mean ± standard deviation (SD). Statistical analysis was performed using GraphPad Prism. Significance levels compared to the control group are indicated as follows: *p* ≤ 0.0001 (****).

Mitochondrial dysfunction is a major contributor to muscle degeneration in sarcopenia; therefore, the mitochondrial membrane potential (MMP) was assessed after 48h of treatment (Figure 11). The Sarco group exhibited a significant loss of MMP compared to the experimental control, indicating marked mitochondrial depolarization and dysfunction. Treatment with DMPC_CAF showed a significant protective effect, maintaining MMP at levels comparable to the experimental control, suggesting a strong mitochondrial stabilizing capacity. In contrast, DOPC_HAMA did not produce a significant improvement, as MMP values remained significantly reduced as compared to the experimental control, similar to those observed in the Sarco group. Notably, the combined DMPC + CAF_DOPC + HAMA formulation fully restored MMP to levels similar to the experimental control, highlighting its enhanced efficacy in preserving mitochondrial integrity under oxidative stress.

**Figure 12:**
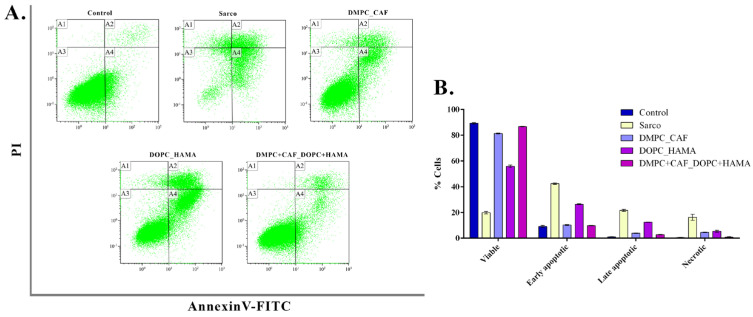
Detection of apoptosis by flow cytometry after labeling with Annexin V FITC/PI in C2C12 myotube cultures after 48 h of treatment with DMPC_CAF, DOPC_HAMA, and the combined DMPC + CAF_DOPC + HAMA formulation. (**A**) Representative flow cytometry dot-plots showing annexin V(^−^)/PI(^−^) viable cells in the lower left quadrant, annexin V(^+^)/PI(^−^) early apoptotic cells in the lower right quadrant, annexin V(^+^)/PI(^+^) late apoptotic cells in the upper right quadrant, and annexin V(^−^)/PI(^+^) necrotic cells in the upper left quadrant. (**B**) Graphical representation of the distribution of the cells (% cell percentage) between viable, early apoptotic, late apoptotic, and necrotic cells after 48 h of treatment. Except for the experimental control (untreated C2C12 myotubes), all groups were exposed to H_2_O_2_ to induce a sarcopenia-like phenotype.

To further assess apoptotic damage induced by oxidative stress in C2C12 myotubes, flow cytometry was performed following Annexin V FITC/PI staining to distinguish between viable, apoptotic, and necrotic cell populations (Figure 12). The Sarco group showed a pronounced increase in early apoptotic (±42.4%), late apoptotic (±21.57%), and necrotic (±16.2%) populations compared with the experimental control, accompanied by a statistically significant reduction in viable cells (±19.82%) compared to the untreated control (±89.31%). Treatment with DMPC_CAF markedly reduced early (±10.23%) and late apoptotic (±3.89%) populations, restoring the proportion of viable cells to ±81.35%, a level comparable to that of experimental control cultures. DOPC_HAMA treatment led to a moderate reduction in apoptotic and necrotic populations, though significantly less pronounced than DMPC_CAF, with viable cells reaching only ±55.81%, representing a 1.6-fold decrease compared to the control sample. All apoptotic and necrotic subpopulations in this group remained significantly altered relative to the untreated control. In contrast, the DMPC + CAF_DOPC + HAMA co-treatment demonstrated the most substantial protective effect, with a high proportion of viable cells (±86.64%) and significantly reduced apoptotic and necrotic populations, closely mirroring the cell subpopulation profile of the control sample. These findings suggest that the combined liposomal formulation exerts a potent anti-apoptotic and cytoprotective effect, likely due to a synergistic interaction between caffeine and HAMA.

## 3. Discussion

Sarcopenia is a concerning degenerative condition characterized by the progressive decline of skeletal muscle mass, function, and strength. As evidenced in recent studies, its onset is closely associated with a series of biological disruptions, including a decrease in both the number and regenerative function of satellite cells, the presence of chronic low-grade inflammation, and elevated oxidative stress levels [43,44]. Modern biomedical research enables a more refined understanding of these degenerative mechanisms and supports the development of targeted therapeutic interventions.

In this context, the current study explores a novel therapeutic approach based on the design and evaluation of liposomal controlled release systems encapsulating two bioactive agents: caffeine and hyaluronic acid. Liposomes are particularly well-suited for this application due to their capacity to restore compromised cell membranes subjected to oxidative stress and to protect the encapsulated agents from degradation. These systems allow for the gradual and controlled release of therapeutic compounds into the targeted environment, where the local biochemical balance may be partially restored upon interaction with the liposomal vesicles [45].

Nevertheless, the physicochemical properties of liposomal formulations are critical determinants of their biological performance. Once administered, these delivery systems interact with plasma biomolecules, including phagocytic immune cells, which may recognize and eliminate them as foreign entities. Moreover, this recognition can initiate downstream inflammatory responses, posing additional challenges for efficient drug delivery [46]. The Food and Drug Administration (FDA) has outlined a set of physicochemical parameters that liposomal formulations must meet to be considered suitable for therapeutic use. These include particle size, polydispersity index, zeta potential, morphology, encapsulation efficiency, bilayer composition, and thermal stability [47]. Among these, the PDI should ideally not exceed 0.3, while the optimal size range for liposomes intended for clinical applications lies between 50 and 200 nm. These characteristics ensure uniformity, efficient biodistribution, and favorable interactions with biological membranes.

All the liposomal formulations developed in this study, DMPC_Ctrl, DMPC_CAF, DOPC_Ctrl, DOPC_HAMA, and the hybrid DMPC + CAF_DOPC + HAMA, displayed PDI values of 0.2, well within the recommended range, indicating a monodisperse and stable vesicle population suitable for therapeutic delivery. Additionally, the measured diameters of all liposomes were included within the 50–200 nm interval, supporting their suitability for prolonged systemic circulation. Regarding surface charge, which is assessed through zeta potential, values within the range of +16 to +55 mV have been associated with favorable colloidal stability in liposomal systems. However, negatively charged liposomes are often considered less desirable in systemic applications due to their tendency to adsorb plasma proteins, which can trigger immune recognition and rapid clearance from circulation [48]. The zeta potential analysis of the developed liposomal formulations revealed that all samples exhibited negative surface charges, but with values within the stability range reported in the literature. Notably, the DOPC_HAMA formulation presented a zeta potential of −16.94 mV, while the hybrid formulation (DMPC + CAF_DOPC + HAMA) showed a more negative value of −28.32 mV, indicating enhanced colloidal stability. This suggests that the combination of DMPC_CAF and DOPC_HAMA liposomes contributes to a synergistic improvement in the stability of the resulting suspension.

Regarding physicochemical parameters influencing liposomal clearance, it is well-established that larger liposomes are eliminated more rapidly from systemic circulation. As such, small unilamellar vesicles are preferred over giant multilamellar vesicles due to their longer circulation time, lower clearance rates, and superior ability to traverse biological membranes, which enhances drug delivery efficiency [47]. While negatively charged liposomes are generally cleared more readily due to plasma protein adsorption, this feature may prove beneficial in the specific context of sarcopenia. The adsorption of certain plasma proteins onto the liposome surface can facilitate targeted accumulation in inflamed or regenerating skeletal muscle tissue. Moreover, using small unilamellar vesicles adds a pharmacokinetic advantage, as their smaller size supports deeper tissue penetration and more efficient cellular uptake. Thus, combining a negatively charged surface and using size-optimized liposomes may synergistically enhance therapeutic retention, target specificity, and biological efficacy for treating muscle degeneration. Furthermore, it is important to note that even when liposomes are surface-modified with polyethylene glycol (PEG), the most common strategy for reducing immune recognition, plasma protein adsorption still occurs, as demonstrated in previous studies [48,49].

Liposomes are spherical vesicles composed of one or more phospholipid bilayers, structurally analogous to biological membranes, and characterized by uniform, defect-free morphology [50,51,52]. The morphology of liposomes plays a critical role in determining their biological behavior, including cellular uptake, circulation time, and overall pharmacokinetic profile. Among various shapes, the spherical vesicular form is widely regarded as optimal for ensuring efficient drug delivery and biodistribution [53,54,55]. The results obtained in this study confirm that all tested liposomal formulations display a well-defined spherical morphology and intact membrane structure, as observed by SEM analysis. These findings are consistent with the literature and support the expectation that such morphological characteristics contribute positively to the biological performance and therapeutic efficacy of the liposomal systems investigated.

All the physicochemical properties of liposomes are intrinsically linked to their drug release kinetics, directly influencing the therapeutic efficacy of the encapsulated agents. In this study, the DMPC-based and DOPC-based formulations exhibited distinct release profiles for their respective active substances, CAF and HAMA. Specifically, the DMPC_CAF formulation demonstrated a gradual, sustained release of CAF over time, aligning with the desired profile for controlled drug delivery. In contrast, the DOPC_HAMA formulation exhibited a burst release effect, characterized by a rapid release of HAMA in the early stages, which may be advantageous for the initial modulation of the extracellular environment, especially given the multifunctional regenerative roles of hyaluronic acid in damaged tissues. The hybrid formulation (DMPC + CAF_DOPC + HAMA) retained the individual release characteristics of both CAF and HAMA, enabling their simultaneous but independent co-release without significant interference. The differences observed in EE% among the formulations, ranging from 30–47%, directly influence their respective drug release behaviors. For instance, the higher EE% of HAMA in the hybrid system (~47%) corresponds with a more pronounced burst release profile, while CAF-loaded DMPC liposomes, with a moderate EE% (~39.6–40.6%), demonstrated a slower, sustained release. This behavior suggests a functional synergy in which HAMA can act rapidly to restore extracellular matrix integrity while CAF provides longer-term metabolic and anti-inflammatory support.

Given that hyaluronic acid is a key component of the extracellular matrix, it may help initiate a regenerative microenvironment in degenerative muscle tissue. Multiple studies have explored the application of hyaluronic acid in muscle repair and regeneration, often in the form of hydrogels or scaffolds, highlighting its biocompatibility and skeletal muscle regeneration potential. A strong precedent supporting the regenerative potential of hyaluronic acid (HA)-based delivery systems is provided by Desiderio et al. [56], who demonstrated that NG2^+^ adipose-derived stem cells, when seeded onto a crosslinked HA scaffold, were capable of successfully differentiating into human skeletal muscle tissue following subcutaneous implantation in nude mice. After 30 days, the materials led to the formation of new myofibers, exhibiting both histological and molecular markers consistent with mature skeletal muscle, thereby confirming the myo-inductive properties of the HA matrix. Similarly, Naagarajan et al. [57] investigated the development of a hyaluronic acid–chitosan hydrogel engineered to support skeletal muscle regeneration. In this study, C2C12 myoblasts were embedded within the hydrogel structure and evaluated for viability, proliferation, and differentiation capacity. The in vitro results showed high levels of cell proliferation and myogenic differentiation, while in vivo studies conducted on murine models with volumetric muscle loss demonstrated the formation of new myofibers in the quadriceps muscle four weeks post-implantation. These findings confirm the therapeutic efficacy of HA-based biomaterials as active participants in muscle tissue repair and regeneration.

In parallel, CAF, as incorporated into the liposomal formulations developed and tested in this study, demonstrated a controlled release profile, reinforcing its suitability for use in sarcopenia therapy. The selection of CAF was based on a growing body of evidence highlighting its metabolic, anti-inflammatory, and myogenic benefits in skeletal muscle. Tsuda et al. [58] investigated the metabolic impact of caffeine on the contracting rat epitrochlearis muscle and found that caffeine significantly enhances the metabolic response to muscle contraction by promoting the activation of essential energy production pathways. Complementing these findings, Yamada et al. [59] demonstrated that caffeine stimulates mitochondrial biogenesis and regulates glucose metabolism in skeletal muscle, effectively replicating the molecular responses observed during physical exercise. Additionally, a large-scale population study conducted by Zhou et al. [44] reported a positive association between dietary caffeine intake and skeletal muscle mass in young adults, suggesting that caffeine may play a role in preventing or delaying the onset of sarcopenia. Further reinforcing this, Guo et al. [30] explored the effects of caffeine on sarcopenia progression through both in vitro and in vivo models. In vitro, caffeine promoted satellite cell proliferation in aged mice compared to untreated controls, while in vivo, it resulted in reduced inflammation, enhanced muscle regeneration, and an increase in muscle mass in aged animal models. Collectively, these findings justify the use of caffeine as a bioactive compound in liposomal formulations targeting sarcopenia, due to its ability to modulate cellular energy metabolism, stimulate muscle regeneration, and mitigate inflammation.

To validate the therapeutic relevance of the liposomal formulations beyond their physicochemical and release characteristics, multiple biological assays were conducted on C2C12 myotube cultures exposed to sarcopenic-like oxidative conditions. In this context, to evaluate the protective effects of the liposomal systems under oxidative stress conditions mimicking the sarcopenic environment, C2C12 myotubes were treated with H_2_O_2_. This condition triggered a reduction in cell viability, elevated ROS production, severe mitochondrial dysfunction, and increased apoptosis [60,61].

Regarding the tested treatments, DMPC_CAF significantly restored viability levels, while DOPC_HAMA showed only a moderate effect. Notably, the DMPC + CAF_DOPC + HAMA treatment achieved the highest protective effect, nearly restoring cell viability to baseline levels. These findings align with reports indicating that CAF enhances cellular resistance to oxidative damage, mainly by activating antioxidant defense pathways and promoting mitochondrial energy metabolism [62,63]. Meanwhile, HAMA supports ECM remodeling, which is critical for muscle integrity and recovery, although it is clear that its effects are more pronounced in tissue-level remodeling than acute cytoprotection. Regarding ROS levels, DMPC_CAF significantly reduced ROS accumulation, which is in agreement with studies showing the key role of caffeine as ROS-scavenging [62,63]. In contrast, DOPC_HAMA offered a minimal reduction in ROS levels, highlighting its limited role in directly modulating oxidative stress. However, the combinatory treatment synergistically lowered ROS levels more effectively than either compound alone, suggesting a dual mechanism of the hybrid formulation.

Furthermore, a similar pattern was observed while assessing the potential of the liposomes to improve mitochondrial function. DMPC_CAF treatment preserved MMP close to control levels, underscoring the role of CAF in supporting mitochondrial bioenergetics and biogenesis [59]. DOPC_HAMA did not significantly improve MMP, further suggesting that its protective role does not extend directly to mitochondria. However, the combined formulation fully restored MMP, indicating a synergistic stabilizing effect.

Finally, the apoptotic status, revealed by Annexin V FITC/PI flow cytometry, included the proportion of viable, apoptotic, and necrotic cell subpopulations under treatment. Treatment with DMPC_CAF significantly reduced both early and late apoptosis, supporting the role of CAF as an antioxidant and mitochondrial stabilization. Interestingly, although caffeine has been reported to induce apoptosis in various cell types [64,65], particularly at high concentrations or in oncological contexts, our findings demonstrate the opposite effect in C2C12 myotubes under oxidative stress. This contrast is likely due to the controlled-release nature of the DMPC liposomal delivery, which ensures gradual cellular uptake and avoids cytotoxic peak concentrations. Moreover, under oxidative stress conditions, CAF may shift from a pro-apoptotic to an anti-apoptotic agent by supporting mitochondrial function, reducing ROS accumulation, and enhancing cell survival pathways. DOPC_HAMA offered moderate protection from apoptosis, which can be attributed to the capacity of HAMA to interact with CD44 and inhibit inflammatory signaling [66]. Nevertheless, the hybrid treatment reduced all apoptotic and necrotic populations to near-control levels, highlighting a robust anti-apoptotic and cytoprotective synergy when both bioactive agents are co-delivered through optimized liposomal carriers.

Overall, the results validate the hypothesis that dual bioactive liposomal delivery can efficiently address the multifaceted cellular dysfunctions underlying sarcopenia. CAF, encapsulated in DMPC vesicles, offers potent intracellular protection by reducing ROS levels, maintaining MMP, and preventing apoptosis. HAMA, encapsulated in DOPC vesicles, complements the effects of CAF by modulating the extracellular environment and potentially enhancing cell–ECM communication, which is essential for long-term regeneration signaling and tissue integrity. Overall, their co-delivery in a hybrid vesicle offers a unique therapeutic strategy that aligns with the complexity of sarcopenic degeneration.

## 4. Materials and Methods

### 4.1. Materials

The synthesis plan incorporates the following materials: 1,2-dimyristoyl-sn-glycero-3-phosphocholine (DMPC), 1,2-dioleoyl-sn-glycero-3-phosphocholine (DOPC), chloroform (CHCl_3_), caffeine (CAF, C_8_H_10_N_4_O_2_), hyaluronic acid methacrylate (HAMA) (NaC_20_H_28_NO_15_)_n_), and ultrapure water. Both DMPC and DOPC were sourced from Avanti Polar Lipids (Sigma Aldrich/Merck, Burlington, MA, USA). Chloroform and HAMA were also obtained from Sigma Aldrich/Merck (Burlington, MA, USA). The caffeine used in this study was sourced from a commercially available supplement (OstroVit, Zambrów, Poland), marketed as pure caffeine powder. Although OstroVit is a recognized European supplement brand, using a dietary supplement rather than pharmaceutical-grade or analytical-grade caffeine represents a methodological limitation. Potential batch-to-batch variability and lack of a certificate of analysis may affect the reproducibility and precise quantification of the active compound.

### 4.2. Methods

The synthesis of liposomes involved using two types of lipids—DMPC and DOPC—to separately encapsulate caffeine and HAMA. These two types of liposomes were subsequently combined to enhance the therapeutic potential of the proposed delivery system.

In the first phase, lipid vesicles based on DMPC encapsulating caffeine were prepared. A thin lipid film was formed by dissolving 8 mg of DMPC in 32 mL of chloroform (1:4 *w*/*v*) and evaporating the solvent using a rotary evaporator under the following conditions: 42 °C, 65 RPM, and 500 mbar pressure. The resulting film was hydrated with ultrapure water and subjected to an ultrasonic bath for one hour. To obtain small unilamellar vesicles, the dispersion was then sonicated using an ultrasound probe set at 20% amplitude for 5 min, with 10 s on/off cycles. For the control sample (DMPC_Ctrl), 10 mL of the hydrated DMPC stock solution was mixed with 20 mL of ultrapure water and then sonicated under the same conditions, resulting in a clear liposomal suspension. The caffeine-loaded sample (DMPC_CAF) was prepared following the same protocol, with caffeine dissolved in ultrapure water, yielding a final concentration of 5 mg/mL in the liposomes. Similarly, DOPC-based liposomes encapsulating HAMA were synthesized using the same procedure. A control sample (DOPC_Ctrl) and a HAMA-loaded sample (DOPC_HAMA) with a final concentration of 1 mg/mL were obtained. Finally, the therapeutic formulation was prepared by mixing equal volumes (1:1 *v*/*v*) of the caffeine-loaded DMPC liposomes (DMPC_CAF) and the HAMA-loaded DOPC liposomes (DOPC_HAMA), resulting in the composite sample labeled DMPC + CAF_DOPC + HAMA.

### 4.3. Characterization and Evaluation of Liposomes

Developing liposomes as drug delivery systems required a comprehensive analysis of their suspension characteristics, performed using Dynamic Light Scattering (DLS) via the DelsaMax Pro system (Beckman Coulter, Brea, CA, USA). Each sample was prepared using water as the solvent and individually injected into the measurement cell. For each formulation, three replicates were conducted to determine the hydrodynamic diameter, zeta potential, and polydispersity index (PDI) of the liposomes.

To assess the morphology and physical dimensions of the liposomes, scanning electron microscopy (SEM) was employed. A volume of 10 µL from each sample was pipetted onto a double-sided carbon adhesive on the sample holder and allowed to dry gradually at room temperature (22–24 °C) under contamination-free conditions within the laminar flow hood. No additional vacuum or chemical drying methods were applied prior to SEM analysis. After solvent evaporation, samples were analyzed using the Versa 3D FIB-SEM system (Thermo Fisher Scientific, Waltham, MA, USA). Secondary electron (SE) micrographs were acquired at an accelerating voltage of 10 keV, utilizing the Everhart–Thornley Detector (ETD) to visualize surface morphology.

Following dimensional and morphological assessment, the liposomal formulations were evaluated for two key performance parameters: encapsulation efficiency (EE%) and drug release profile under conditions simulating the physiological environment.

To determine encapsulation efficiency, samples were centrifuged at 8500 RPM for 2 h, enabling the separation of liposome-encapsulated drugs from unencapsulated residual compounds. The supernatant, containing the unencapsulated part, was collected and analyzed using UV-Vis spectroscopy on a Thermo Fisher Scientific Evolution 300 double-beam spectrophotometer (Waltham, MA, USA). Prior to analysis, calibration curves were constructed for both caffeine (CAF) and hyaluronic acid methacrylate (HAMA). Five calibration points were prepared for each substance, ranging from 0.5 to 4 mg/mL for CAF and 0.1 to 0.8 mg/mL for HAMA. Spectral scans were recorded between 190 and 350 nm, with absorption maxima observed at 272 nm for CAF and 210 nm for HAMA. Thus, by processing the data using VisionPro software (version 4.5.0) and by applying the formula for evaluating the encapsulation efficiency, this property of the liposomes encapsulating CAF and HAMA was established. The formula applied is:

$$EE\%=\left(\frac{C_{total}-C_{free}}{C_{total}}\right)\times 100$$

$C_{total}$ refers to the initial concentration of the drug introduced into the system, whereas $C_{free}$ represents the concentration of the drug that remains unencapsulated in the supernatant.

After separating the lipid vesicles from the unencapsulated drug, the pellets from each of the three formulations were redispersed in phosphate-buffered saline (PBS) and incubated in a water bath at 37 °C for 8 h to mimic sustained release conditions in order to evaluate the time-dependent drug release rate. Samples were collected at specific time intervals of 5, 30, 60, 180, 300, and 480 min. At each release interval, 1 mL of sample was collected and diluted 1:1 by mixing with 1 mL of PBS. To ensure uniformity in sample volume and testing conditions, an equal amount of fresh PBS was added to the remaining dispersion after each sampling. The quantification of drug release was performed using UV-Vis spectrophotometry, utilizing the same instrumentation and calibration curves previously established for calculating encapsulation efficiency. The spectral data obtained were used to create the drug release profiles for the three formulations being tested, calculated according to the following equation:

$$Drug\;release\;\%=\left(\frac{C_{release}\;}{C_{encapsulated}}\right)\times 100$$

where $C_{release}$ denotes the concentration of drug released at a given time point, and $C_{encapsulated}$ refers to the total concentration of drug initially encapsulated within the liposomes.

### 4.4. In Vitro Biological Assays

C2C12 murine myoblasts (ATCC CRL-1722) were used to develop an in vitro model of sarcopenia. Cells were maintained initially in Dulbecco’s Modified Eagle’s Medium (DMEM, Sigma-Aldrich), supplemented with 10% fetal bovine serum (FBS, Gibco, Thermo Fisher Scientific, Waltham, MA, USA) and 1% antibiotic–antimycotic solution (ABAM, Sigma-Aldrich, St. Louis, MO, USA). Cultures were maintained at 37 °C in a humidified 5% CO_2_ incubator. Cells were seeded in appropriate culture plates for the proposed assays and allowed to proliferate under the above conditions. Upon reaching approximately 80% confluence, differentiation into myotubes was induced by switching the culture medium to high-glucose DMEM supplemented with 2% horse serum (HS, Sigma-Aldrich). After 4 days of differentiation, cells were randomly assigned to the following groups: (i) control (differentiation medium only); (ii) sarcopenia model (100 μM H_2_O_2_ to induce oxidative stress), and (iii) treatment groups (100 μM H_2_O_2_ co-administered with DMPC_CAF, DOPC_HAMA, and the combined DMPC + CAF_DOPC + HAMA formulation). All conditions were maintained for 48 h.

Cell viability was evaluated using the MTT assay (3-(4,5-Dimethylthiazol-2-yl)-2,5-Diphenyltetrazolium Bromide, Sigma-Aldrich). Following 48h of treatment, the culture medium was replaced with 1 mg/mL MTT prepared in serum-free DMEM, and cells were incubated for 4 h at 37 °C. The resulting formazan crystals were dissolved in isopropanol, and absorbance was measured at 550 nm using a FlexStation 3 microplate multimodal reader (Molecular Devices, San Jose, CA, USA). Viability was calculated relative to the untreated control as follows: Cell viability (%) = (Absorbance of treated cells/Absorbance of control cells) × 100.

Intracellular ROS levels were assessed using the fluorescent probe DCFH-DA (2′, 7′-dichlorofluorescein diacetate, Sigma-Aldrich). After 48 h of treatment, cells were washed with PBS and incubated with 10 μM DCFH-DA in serum-free DMEM for 30 min at 37 °C in the dark. Excess dye was removed by PBS washing, and fluorescence was measured immediately at excitation/emission wavelengths of 485/530 nm using a FlexStation 3 microplate reader (Molecular Devices). ROS levels were expressed relative to the untreated control: ROS Production (%) = (Fluorescence of treated cells/Fluorescence of control cells) × 100.

Mitochondrial membrane potential (MMP) was evaluated using the JC-10 dye provided in the Mitochondrial Membrane Potential Kit (MAK159, Sigma-Aldrich). Cells were incubated for 45 min at 37 °C in the dark with 50 µL of JC-10 Dye Loading Solution, prepared by diluting the 100X JC-10 stock in Assay Buffer A. Following incubation, 50 µL of Assay Buffer B was added to each well. Fluorescence was then measured using a FlexStation 3 microplate reader (Molecular Devices) at λex = 540 nm and λem = 590 nm for JC-10 aggregates (red fluorescence, polarized mitochondria), and λex = 490 nm and λem = 525 nm for JC-10 monomers (green fluorescence, depolarized mitochondria). The red/green fluorescence ratio was calculated, and results were normalized to control levels (set as 100%) to express MMP changes as a percentage.

Apoptosis was quantified using the Dead Cell Apoptosis Kit with Annexin V FITC and Propidium Iodide for Flow Cytometry (Invitrogen, Carlsbad, CA, USA). After 48 h of treatment, cells were harvested, centrifuged (1500 rpm, 5 min), and washed with PBS. Cell pellets were resuspended in 1X annexin-binding buffer at ∼1 × 10^6^ cells/mL. Each 100 μL of the sample was stained with 5 µL Annexin V FITC and 1 µL PI (100 µg/mL), incubated for 15 min at room temperature in the dark and diluted in the end with 400 µL of 1X annexin-binding buffer. Samples were immediately analyzed by flow cytometry using a Gallios cytometer (Beckman Coulter, Brea, CA, USA), detecting FITC (FL1) and PI (FL3). Data were analyzed using Kaluza v2.1 software (Beckman Coulter).

All experiments were performed in biological triplicates. Data are presented as mean ± standard deviation (SD). Statistical significance was evaluated using GraphPad Prism, with the statistical significance threshold determined at *p* < 0.05

## 5. Conclusions

In this study, a liposomal controlled release system was developed and evaluated for its potential to improve and therapeutically support sarcopenia, by addressing key biological deficiencies and degenerative processes associated with skeletal muscle deterioration. Two distinct liposomal systems were engineered: one based on DMPC vesicles encapsulating caffeine (CAF) and another based on DOPC vesicles encapsulating hyaluronic acid methacrylate (HAMA). Subsequently, a hybrid formulation was created by combining both types of liposomes, each containing its respective bioactive compounds. A comprehensive physicochemical characterization was conducted following the evaluation criteria established by the Food and Drug Administration (FDA) for liposomal products. The results confirmed that all formulations exhibited nanometric particle sizes, a polydispersity index (PDI) of 0.2, and colloidal stability, as indicated by zeta potential values ranging from moderate to high. Structural analysis via scanning electron microscopy (SEM) revealed that the liposomes possessed spherical morphology, well-defined edges, and no detectable structural deformities, confirming the successful formation of uniform vesicular architectures. Differences in encapsulation efficiency were observed between the two systems, attributed to the distinct chemical properties of the lipid matrices and active agents.

Furthermore, drug release studies in a physiologically simulated environment demonstrated that each formulation displayed a unique release profile over time. Of particular note, the hybrid system exhibited a dual functionality tailored for the targeted application: HAMA showed a more rapid initial release, beneficial for early extracellular matrix modulation, while CAF demonstrated a slower, sustained release, supporting long-term metabolic and anti-inflammatory action. These results support the suitability of the hybrid liposomal system for co-delivery of bioactive compounds with complementary therapeutic roles, as required in multifactorial conditions such as sarcopenia. The biological assays performed on an in vitro cellular model of sarcopenia demonstrated that the liposomes encapsulating CAF and HAMA offer a multifaceted protective effect against sarcopenia-like cellular damage in muscle cells. By simultaneously addressing oxidative stress, mitochondrial dysfunction, and apoptotic cell death, this combinatorial strategy holds great promise as a novel therapeutic avenue for sarcopenia management. Although the biological assays demonstrated significant protective effects at the cellular level, future studies should aim to investigate the specific downstream molecular pathways involved in order to complement these functional observations. Ongoing research and in vivo studies are necessary to confirm the clinical applicability of our findings.

## Figures and Tables

**Table 1:** Data values obtained for the hydrodynamic diameter of DLS measurements of DMPC_Ctrl, DMPC_CAF, DOPC_Ctrl, DOPC_HAMA, and DMPC + CAF_DOPC + HAMA.

	DMPC_Ctrl	DMPC_CAF	DOPC_Ctrl	DOPC_HAMA	DMPC + CAF_DOPC + HAMA
Hydrodynamic diameter (nm)	164.63	230.53	116.5	166.2	146.53

**Table 2:** Data values obtained for zeta potential of DLS measurements of DMPC_Ctrl, DMPC_CAF, DOPC_Ctrl, DOPC_HAMA, and DMPC + CAF_DOPC + HAMA.

	DMPC_Ctrl	DMPC_CAF	DOPC_Ctrl	DOPC_HAMA	DMPC + CAF_DOPC + HAMA
Zeta potential (mV)	−13.7	−10.42	−13.10	−16.94	−28.32

**Table 3:** Data values obtained for polydispersity index (PDI) of DLS measurements of DMPC_Ctrl, DMPC_CAF, DOPC_Ctrl, DOPC_HAMA, and DMPC + CAF_DOPC + HAMA.

	DMPC_Ctrl	DMPC_CAF	DOPC_Ctrl	DOPC_HAMA	DMPC + CAF_DOPC + HAMA
PDI	0.2	0.2	0.2	0.2	0.2

## Data Availability

The data presented in this study are available on request from the corresponding author. The data are not publicly available due to their nature and format.
